# Clinical and Patient-Related Variables Associated with Initiating GLP-1 Receptor Agonist Therapy in Type 2 Diabetes Patients in Primary Care in Germany

**DOI:** 10.1371/journal.pone.0152281

**Published:** 2016-03-28

**Authors:** Qing Qiao, Susan Grandy, Josh Hiller, Karel Kostev

**Affiliations:** 1 AstraZeneca, Global Medicines Development, Mölndal, Sweden; 2 AstraZeneca, Global Medicines Development, Gaithersburg, Maryland, United States of America; 3 IMS Health, Real World Evidence Solutions, London, United Kingdom; 4 IMS Health, Epidemiology, Frankfurt am Main, Germany; University of Lancaster, UNITED KINGDOM

## Abstract

**Aims:**

To investigate real-world clinical and patient-related variables associated with initiating GLP-1 receptor agonist (GLP-1RA) treatment relative to initiation of other glucose-lowering therapies in type 2 diabetes (T2D) patients of primary care in Germany.

**Methods:**

Data for 938 T2D patients who started therapy with a GLP-1RA within 823 practices of primary care throughout Germany were retrospectively analyzed (Disease Analyser: 01/2011–03/2014). 5,197 T2D patients who initiated other non-GLP-1RA antidiabetic therapies were selected as controls. Multivariate logistic regression analyses were applied to identify factors associated with GLP-1RA initiation in primary care.

**Results:**

Mean age (SD) of GLP-1RA users was 57.8 (11.8) years (males: 55.5%) and the average BMI was 36.1 (6.7) kg/m^2^. 22.8% were in diabetologist care and 12.0% had private health insurance. In multivariate regression, choice of GLP-1RA therapy instead of a different glucose-lowering drug class was associated with obesity (odds ratio: 1.68; 95% CI: 1.34–2.10), private health insurance (2.42; 1.89–3.09), younger age (0.94; 0.93–0.95 per year), male sex (0.85; 0.73–0.99), diabetologist care (2.11; 1.73–2.57), and geographic practice location (East vs. West-Germany; 1.25; 1.05–1.49). Among co-medication, angiotensin II antagonists (increased) and non-steroidal antirheumatic agents (decreased) were related to GLP-1RA prescriptions (both p<0.001).

**Conclusions:**

Consistent with German guidelines, GLP-1RA is mainly prescribed preferentially in T2D patients who are obese. GLP-1RA drugs were more frequently used than other options in privately health insured patients and in patients seeing a diabetologist.

## Introduction

Metformin is recommended as first line drug treatment for type 2 diabetes both in the German National Disease Management Guideline on the Treatment of Type 2 Diabetes, and the guidelines of the American Diabetes Association (ADA) and the European Association for the Study of Diabetes (EASD) [1–3]. These guidelines recommend GLP-1 receptor agonists (GLP-1RAs) as add-on treatment to metformin if hyperglycemia is still not sufficiently controlled with metformin alone [1–3]. GLP-1RAs increase insulin secretion and inhibit glucagon release, but only in the presence of elevated glucose levels [4]. This mode of action is different from sulfonylureas and glinides, which are associated with higher risk of hypoglycemia because they increase insulin secretion regardless of the actual glucose levels. Hypoglycemia is also a disadvantage of insulin therapy. Furthermore, insulin therapy in type 2 diabetes is often associated with weight gain [5], whereas GLP-1RA treatment often results in weight loss [6].

Little information is available about patient-related characteristics and other clinical factors leading to initiation of GLP-1RA therapy instead of other non-GLP-1RA antidiabetic agents in real-world primary care settings. Few studies have examined the initiation of GLP-1 RA therapy in type 2 diabetes patients in a real-world setting [7–10]. As an example in the UK, compared with insulin starters, those initiating a GLP-1RA therapy had higher body mass index (BMI) and better glycaemic control at baseline, and were younger with shorter duration of diabetes [7]. The objective of this study was to identify clinical and patient-related variables associated with initiating GLP-1 RA therapy in type 2 diabetes patients in real-world primary care settings in Germany.

## Methods

The Disease Analyzer database (IMS HEALTH) assembles drug prescriptions, diagnoses, and basic medical and demographic data directly obtained from the computer system of a representative sample of primary care clinics of general practitioners and diabetologists throughout Germany [11]. The database includes only anonymized data in compliance with the regulations of the applicable data protection laws. All patient records/information are anonymized prior to including in the database. In Germany, studies based on such anonymized databases do not need ethical approval. The analyzed database period for the current study was from January 2011 to March 2014 (823 primary care clinics). Patients with type 2 diabetes, who were not prescribed with a GLP-1RA in the 6 months pre-index period, but initiated on a GLP-1RA (index date: exenatide BID, exenatide EQW or liraglutide) during the study period, were identified as GLP-1RA initiator cohort. The comparison cohort consists of type 2 diabetes patients who were not prescribed a GLP-1RA in the 6 months pre-index period but on first line anti-diabetic drug treatment in the pre-index period, who subsequently had new prescriptions of other non-GLP1-RA antidiabetic agents (oral antidiabetic drugs or insulin that were different from the drug used in the pre-index period) on the index dates during the same study period.

Baseline characteristics of a patient were determined based on the patient records made by a physician during the 6-months pre-index period. Potential predictors of GLP-1RA initiation considered in the present analysis were age, sex, type of health insurance, region of practice (East or West-Germany), diabetologist care, glycemic control (HbA1c), comorbidity (cardiovascular and renal diseases, microvascular diabetes complications, mental disorders) and co-medications. Gender was subsequently explored in more detail due to findings of interest.

Macrovascular complications were determined based on diagnoses (ICD-10 codes) for coronary heart disease (I24, I25), myocardial infarction (I21, I22, I23, I25.2), stroke (I63, I64, G45), peripheral vascular disease (I739, E105, E115, E145) and heart failure (I50). Microvascular complications included retinopathy (E113, E143, H360), neuropathy (E114, E144), and nephropathy (N18, N19, E112, E142, Z49, Z992). Presence of mental disorders including depression and dementia (F20-F48) was also assessed. Furthermore, diagnosed obesity (ICD 10: E66), lipid disorders and hypertension were considered as covariables. Medication with cardiovascular and lipid-lowering drugs during the 6-months pre-index period was also assessed. Finally, the recorded HbA1c values and the documented BMI before index date were included in the analyses. The Charlson co-morbidity index was used as a general marker of co-morbidity. The Charlson index is a weighted index that accounts for the number and severity of co-morbidities in administrative database studies [12].

Descriptive statistics were given and differences in characteristics of patients (GLP-1RA vs. controls) were assessed using *t*-tests, Wilcoxon-tests, or chi-square tests. Two sided tests were used and a p-value of <0.05 without adjustments for multiple comparisons was considered as statistically significant. A final multivariate logistic regression model was fitted using stepwise selection. Variables that did not have statistical significance (p>0.05) were removed stepwise. In addition, sex-specific models were fitted. All analyses were carried out following the German good practice recommendations of secondary data analysis [13] using SAS 9.3 (SAS Institute, Cary, USA).

## Results

After patient selection, 938 new users of GLP-1RAs (mean age: 57.8 years; 55% males) and 5197 type 2 diabetes patients (67.4 years; 55% men) initiating other non-GLP1-RA agents without any GLP-1RA prescriptions at baseline were included. The baseline clinical characteristics are shown in Table 1. Some patients were privately insured and approximately three quarters of all patients were from West Germany. Almost a quarter of the type 2 diabetes patients with GLP-1RA were treated by diabetologists. The mean BMI and the average HbA1c were both high. Biguanides were the most frequently prescribed antidiabetic drug at baseline, followed by DPP-4 inhibitors and sulfonylureas (Table 1). Insulin was used in 16% of the GLP-1RA initiators, either alone or in combination with other agents.

**Table 1:** Baseline characteristics of type 2 diabetes patients who initiated GLP-1 receptor agonists (GLP-1RAs) or other non-GLP-1RA anti-diabetic drugs (Control) between January 2011 and March 2014 in primary care in Germany.

Variables	Men		Women		Total	
Variables	GLP-1 RAs	Control	GLP-1 RAs	Control	GLP-1 RAs	Control
N	521	2838	417	2359	938	5197
Age (years)	57.3 (11.5)*	65.9 (11.9)*	58.4 (12.2)*	69.3 (12.7)*	57.8 (11.8)*	67.4 (12.4)*
Private health insurance (%)	15.9*	8.0*	7.2*	3.8*	12.0*	6.1*
Diabetologist care (%)	23.0*	11.0*	22.5*	10.8*	22.8*	10.9*
Practice region (East Germany) (%)	28.8*	20.4*	23.5	21.5	26.4*	20.9*
HbA1c %	8.2 (1.5)	8.1 (1.6)	8.1 (1.5)	7.9 (1.5)	8.1 (1.5)*	8.0 (1.6)*
Body mass index (kg/m^2^)	35.9 (7.0)*	30.8. (5.8)*	36.4 (6.3)*	31.9 (6.3)*	36.1 (6.7)*	31.3. (6.1)*
Peripheral neuropathy (%)	8.8	8.1	7.9	7.3	8.4	7.8
Retinopathy (%)	2.5	1.7	1.2	1.8	1.9	1.8
Nephropathy (%)	7.1	7.6	6.0	7.5	6.6	7.5
*Baseline diagnoses*:						
Hypertension (%)	50.3*	56.4*	49.6*	61.1*	50.0*	58.6*
Dyslipidemia (%)	26.9	30.7	25.2	27.3	26.1	29.1
Obesity diagnosis (%)	14.2*	6.8*	17.7*	7.4*	15.8*	7.0*
Myocardial infarction (%)	1.5	2.9	0.7	1.1	1.2	2.1
Coronary heart disease (%)	9.6*	18.3*	9.8	12.0	9.7*	15.4*
Peripheral vascular disease (%)	3.5*	7.1*	4.3	4.5	3.8*	5.9*
Mental illness (%)	11.5	12.6	19.9	20.9	15.2	16.3
Charlson Comorbidity Score	1.9 (1.5)	1.9 (1.4)	1.7 (1.1)	1.9 (1.5)	1.8 (1.3)	1.9 (1.5)
*Baseline anti-diabetic medication*:						
Metformin (%)	41.7*	70.3*	46.3*	69.3*	43.7*	69.8*
Sulfonylureas (%)	14.6*	21.1*	12.5*	22.7*	13.6*	21.8*
DPP-4 inhibitors (%)	27.6*	3.3*	24.0*	4.4*	26.0*	3.8*
Insulin (%)	16.5*	2.1*	15.3*	2.1*	16.0*	2.1*
Other OADs (%)	9.9*	5.2*	14.1*	8.0*	9.4*	5.0*
*Baseline medication*:						
Diuretics (%)	18.8*	25.8*	19.2*	32.4*	19.0*	28.8*
Beta-blockers (%)	35.7*	41.8*	33.6*	46.7*	34.8*	44.4*
Calcium channel blockers (%)	18.4*	22.6*	18.0*	26.3*	18.2*	24.3*
ACE inhibitors (%)	33.6*	42.1*	23.7*	39.2*	29.2*	40.8*
Angiotensin II receptor blockers (%)	23.0*	18.5*	26.1*	21.4*	24.4*	19.8*
Lipid lowering drugs (%)	30.9*	38.0*	25.4*	31.7*	28.5*	35.1*
Non-steroidal antirheumatic agents and other analgesics (%)	27.4*	34.5*	33.3*	42.2*	30.1*	38.0*

Data are means (SD) or proportions (%).

* *p-*value <0.05 for the difference between the two treatment groups. Mental illness include schizophrenic, mood and neurotic disorders.

Arterial hypertension was diagnosed in 50% and lipid disorders were found in about a quarter of patients (Table 1). Coronary heart disease was diagnosed in 10% of the patients initiating GLP-1RA treatment. About 1% had already suffered from a myocardial infarction. Peripheral vascular disease was diagnosed in about 4%. Microvascular diabetes complications were also observed, in particular, peripheral neuropathy, which was found in 8% of the new GLP-1RA users (Table 1).

Co-medications with antihypertensives, lipid-lowering drugs (including statins), non-steroidal antirheumatic agents and analgesics were each prescribed to about 30% of patients.

GLP-1RA users were about 10 years younger than non-users (Table 1) (p<0.0001). GLP-1RA users were twice more often privately health insured (p<0.0001) and more frequently treated by diabetologists (p<0.0001). Furthermore, GLP-1RAs were more prescribed in the primary care practices in East-Germany (p<0.0001).

There were also significant differences in some baseline clinical characteristics of the two groups (Table 1). The average baseline HbA1c did not differ between the two treatment groups in either men or women (Table 1), but a small difference was identified in the population as a whole. The mean body mass index (kg/m^2^) at baseline was higher among GLP-1RA users than in non-users (p<0.0001). The prevalence of obesity defined by a baseline BMI value of ≥30.0 kg/m^2^ (p<0.001), as well as the prevalence of obesity identified based on ICD-10 diagnostic codes (p<0.0001) were higher in patients with GLP-1RA therapy than in the comparator group. However, the prevalence of musculoskeletal disorders (ICD 10: M00-M99) was lower among GLP-1RA users (35.5% vs. 43.2%; p<0.0001).

Differences in medication usage were also found. In both groups, the most often prescribed antidiabetic agent at baseline was metformin, which was more often used in the control group, as well as sulfonylureas (Table 1). In contrast, DPP-4 inhibitors and insulin were more often prescribed in patients who started a GLP-1RA therapy. In addition, other oral antidiabetics (thiazolidinediones, glinides, acarbose) were also more often used in GLP-1RA starters. Non-steroid antirheumatic agents and other analgesics were less often prescribed among GLP-1RA users (p<0.0001), consistent with the lower incidence of musculoskeletal disorders.

The prevalence of macrovascular complications at baseline was significantly lower among GLP-1RA users, except for myocardial infarction (Table 1). Arterial hypertension (p < .0001) and dyslipidemia (p = 0.0604) were also less frequently observed in the GLP-1 RA users. In addition, cardiovascular drugs (ACE inhibitors, angiotensin II antagonists, calcium channel blockers, beta blockers, diuretics) and lipid lowering drugs were also less often used among patients with GLP-1RA prescriptions.

After stepwise selection using logistic regression, several clinical and patient-related variables were independently associated with the initiation of GLP-1RA therapy in type 2 diabetes patients (Table 2). Younger age and male gender were associated with GLP-1RA therapy rather than another therapy. Patients with diagnosed obesity had a higher odds of having GLP-1RA prescriptions than a prescription for a different therapy. Furthermore, diabetologist care was related to a higher chance of receiving GLP-1RA therapy than non-GLP-1RA therapy. Among demographic characteristics, patients with private health insurance had an increased odds of having initiation of GLP-1RA therapy compared to type 2 diabetes patients with statutory health insurance. Furthermore, patients treated in primary care practices located in East-Germany were more likely to have newly prescribed GLP-1RA than in West-German practices. Baseline prescriptions of angiotensin II receptor blockers were related to a higher chance of having GLP-1RA therapy. Prescription use of non-steroidal antirheumatic drugs and other analgesics or antipyretics (e.g. salicyclic acid, pyrazolone) were associated with a lower odds of having initiation of GLP-1RA treatment.

**Table 2:** Multivariate adjusted odds ratios (95% confidence intervals) for initiating GLP-1 receptor agonists compared with initiators of non-GLP-1RA anti-diabetic drugs in type 2 diabetes patients of primary care in Germany.

Variables^a^	Men	*P* value	Women	*P* value	Total	*P* value
Age (per year)	0.94 (0.93–0.95)	<0.0001	0.94 (0.93–0.95)	<0.0001	0.94 (0.93–0.95)	<0.0001
Obesity diagnosis (yes/no)	1.59 (1.17–2.17)	0.0032	1.75 (1.27–2.41)	0.0006	1.69 (1.35–2.11)	<0.0001
Private health insurance (yes/no)	2.38 (1.78–3.19)	<0.0001	2.45 (1.55–3.87)	0.0001	2.41 (1.88–3.09)	<0.0001
Diabetologist care (yes/no)	2.25 (1.74–2.91)	<0.0001	2.05 (1.52–2.76)	<0.0001	2.11 (1.73–2.57)	<0.0001
Angiotensin II receptor blocker prescriptions [ATC: C09C, C09D]	1.67 (1.31–2.14)	<0.0001	1.89 (1.45–2.46)	<0.0001	1.76 (1.47–2.11)	<0.0001
Coronary heart disease (yes/no)^b^	0.72 (0.52–0.99)	0.0479	-	-	1.36 (0.95–1.97)	0.0956
Geographic practice location (East vs. West-Germany)	1.43 (1.14–1.80)	0.0018	-	-	1.25 (1.05–1.49)	0.0119

^a^ Full model included: age, gender, geographic practice location, private health insurance, diabetologist care, HbA1c, baseline co-diagnoses (peripheral neuropathy, retinopathy, nephropathy, hypertension, dyslipidemia, obesity diagnosis, myocardial infarction, coronary heart disease, peripheral vascular disease, mental illness), baseline medication (metformin, sulfonylureas, DPP-4 inhibitors, Insulin, other OADs, diuretics, beta-blockers, calcium channel blockers, ACE inhibitors, angiotensin II receptor blockers, lipid lowering drugs, non-steroidal antirheumatic agents and other analgesics)

^b^ Coronary heart disease and geographic practice location entered into the final model in men only

Some of the findings differed by gender (Table 1). The associations of age, obesity, diabetologist care, private health insurance, and angiotensin II receptor blockers were comparable between men and women (Table 2). However, coronary heart disease was related to GLP-1 RA use in men but not in women. Practice location (East/West-Germany) was also related to GLP-1 RA use in men only.

There were no differences between GLP-1 RA and controls in a stratified analysis (East- vs. West German practice location) compared to the total population (data not shown).

## Discussion

Using a large representative primary care database in Germany the present study indicates that GLP-1RA are more likely initiated in obese and younger type 2 diabetes patients compared to patients initiating non-GLP-1RA therapies. GLP-1RA are also more often prescribed in privately health insured patients. Further predictors were treatment by a diabetologist and primary health care practice location in East-Germany. Baseline angiotensin II receptor blocker prescriptions were related to a higher chance of having a GLP-1RA treatment initiated. Nonsteroidal antirheumatic agents were associated with a lower odds of having these drugs started. Finally, coronary heart disease was related to a lower odds of having GLP-1 RA started in men but not in women.

The finding that GLP-1 RA therapy was favored by physicians in patients with higher body weight is not surprising and is in line with previous observational studies [7, 9]. Body weight reduction with GLP-1 RA has been confirmed in a meta-analysis of randomized controlled trials [6]. The mean weight reduction in patients with diabetes was -2.8 kg, which makes GLP-1 RA a preferred option for patients who are obese compared with treatments which may increase weight, including sulfonylureas and insulin [6].

Patients initiating GLP-1 RA were substantially younger than those initiating non-GLP-1RA drugs, which has also been found in previous studies [7,9]. Using retrospective electronic medical records from the General Electric Centricity database in the U.S., factors associated with exenatide initiation were younger age and a body mass index of 35 kg/m^2^ or greater [8]. Based on administrative pharmacy, medical claims, and laboratory result data obtained from the HealthCore Integrated Research Database, type 2 diabetes patients with an initial claim for exenatide or insulin glargine between May 1, 2005 and June 30, 2007 were retrospectively analysed [10]. Cardiovascular diseases, nephropathy, and retinopathy were all more prevalent among patients initiating insulin glargine [10]. This finding is further supported by the CHOICE study [9]. CHOICE was a prospective observational study that recruited patients initiating their first injectable glucose-lowering therapy (with any type of insulin or exenatide) in routine clinical practice from six European countries (Denmark, Belgium, France, Germany, Greece and Sweden) [9]. A higher proportion of patients initiating insulin had microvascular and macrovascular complications compared with patients starting exenatide, which is in line with the present study [9]. Younger age of the GLP-1RA cohort to certain extent explains the lower prevalence of comorbidities in this cohort as compared with the control cohort.

GLP-1 RA therapy was also more often started in patients treated by diabetologists, which most likely reflects the fact that diabetologists are more familiar with the relatively new incretin-based therapies, and that the patients situation most likely requires a specialized diabetologist care.

Furthermore, although the majority of the patients were covered by a statutory health insurance scheme, it is noteworthy that private health insurance was a strong predictor of having GLP-1 RA therapy initiated, independent of other characteristics.

During the last four decades, the proportion of people having full private health insurance cover in Germany rose from 6.9% in 1975 to 11.0% in 2012 [14]. This increasing division into statutory and private health insurance is a challenge and may contribute to health care inequalities [14]. As an example, novel and more expensive glucose-lowering drugs such as GLP-1 RAs, DPP-4 inhibitors and SGLT2 inhibitors are less often prescribed in type 2 diabetes patients with statutory compared with patients with private health insurance [15].

For statutory health insured patients, disease management programs (DMP) for diabetes have been implemented [16]. The DMPs should harmonize diabetes management [16]. As a consequence it can be assumed that a homogenous treatment pattern should exist throughout Germany. However, the present study indicates regional differences in GLP-1 RA use in Germany. Men with type 2 diabetes treated in general practices in East-Germany were more likely to start GLP-1 RAs therapy but this was not observed in women, which needs to be confirmed in a larger study sample. Regional differences in prescription use of newer glucose-lowering drugs have also been recently described in population-based regional health surveys in Germany [17]. Further research is needed to explain these findings.

It was also noted that some of the baseline concomitant medications were either positively or negatively associated with the initiation of GLP-1 RA therapy. That may be related to the comorbidities that patients had at baseline, rather than a contributor directly leading to the prescription of GLP-1RA. A typical example is that male patients who had CHD at baseline were less likely to initiate GLP-1RA, but women had an equal chance to get a prescription of GLP-1RA compared with controls. Since GLP-1RA has no contraindications for patients with cardiovascular diseases, having a cardiovascular disease at baseline may or may not affect a doctor’s decision on initiating GLP-1RA.

The primary objective of this study was to better understand the clinical characteristics of type 2 diabetes patients in primary care initiating GLP-1RA therapy. According to national and international guidelines, GLP-1RA are an option for second-line treatment in patients with insufficient glycemic control with metformin monotherapy. Our study results strongly indicate that prescription use of GLP-1RA by primary care physicians is currently mainly focused on obese and middle-aged type 2 diabetes patients. Thus, primary care physicians limit their spectrum of options for second-line drugs, which is also due to budget constraints (cost-intensive GLP-1RA). They seem to be reluctant to prescribe injectable agents for the majority of patients with uncontrolled type 2 diabetes. This indicates a need for more patient-centered treatment decisions for type 2 diabetes subjects unable to achieve and maintain glycemic targets.

Because the study was based on the primary care medical records, a number of limitations should be mentioned. First, no valid information regarding onset of diabetes was provided. Furthermore, assessment of co-morbidities relied on ICD codes filled in by physicians that may under estimate the prevalence of certain comorbidities such as obesity. Due to privacy protection, data on geographic location of practices is not available. We cannot make further data analysis to check whether the study results have been heavily driven by cluster of prescriptions to GLP-1RAs in certain practices. Since the study results are in line with previous reports we have no reason to speculate the study has been biased by the potential cluster of practices. Finally, measurements of HbA1c and body mass index values were not standardized. The strength of the study is the large nationwide database and the unbiased assessment of prescriptions.

To conclude, in Germany GLP-1 RA has been commonly prescribed to type 2 diabetes patients who were obese, and had a private health insurance coverage. GLP-1RA therapy instead of non-GLP-1RA therapy was more likely to be initiated by a diabetologist than by a general practitioners.

## References

[1] Bundesärztekammer (BÄK) Kassenärztliche Bundesvereinigung (KBV), Arbeitsgemeinschaft der Wissenschaftlichen Medizinischen Fachgesellschaften (AWMF). Nationale Versorgungsleitlinie Therapie des Typ-2-Diabetes-Langfassung, 1st edition, Version 2. 2013 last updated on September 2013. Available: www.versorgungsleitlinien.de/themen/diabetes2/dm2_Therapie. Accessed 2015 Aug 4.

[2] American Diabetes Association. Standards of medical care in diabetes: 2015. Diabetes Care. 2015; 38 (Suppl 1): S1–99.

[3] Inzucchi SE, Bergenstal RM, Buse JB, Diamant M, Ferrannini E, Nauck M, et al Management of hyperglycaemia in type 2 diabetes: a patient-centered approach, Position statement of the American Diabetes Association (ADA) and the European Association for the Study of Diabetes (EASD), Diabetologia, 2012; 55: 1577–96 10.1007/s00125-012-2534-0 22526604

[4] Garber AJ. Long-acting glucagon-like peptide 1 receptor agonists. A review of their efficacy and tolerability. Diabetes Care. 2011; 34 (Suppl 2): S279–84 10.2337/dc11-s231 21525469 PMC3632193

[5] Eng C, Kramer CK, Zinman B, Retnakaran R. Glucagon-like peptide-1 receptor agonist and basal insulin combination treatment for the management of type 2 diabetes: a systematic review and meta-analysis. Lancet. 2014; 384: 2228–34. 10.1016/S0140-6736(14)61335-0 25220191

[6] Vilsboll T, Christensen M, Junker AE, Knop FK, Gluud LL. Effects of glucagon-like peptide-1 receptor agonists on weight loss: systematic review and meta-analyses of randomised controlled trials. BMJ. 2012; 344: d7771 10.1136/bmj.d7771 22236411 PMC3256253

[7] Hall GC, McMahon AD, Dain MP, Wang E, Home PD. Primary-care observationaldatabase study of the efficacy of GLP-1 receptor agonists and insulin in the UK. Diabet Med. 2013; 30: 681–6. 10.1111/dme.12137 23330649 PMC3698690

[8] Hirsch IB, Xu Y, Davis KL, Calingaert B. Patient factors associated with glucagonlike peptide 1 receptor agonist use with and without insulin. Endocr Pract. 2011; 17: 707–16. 10.4158/EP11014.OR 21856602

[9] Matthaei S, Reaney M, Mathieu C, Ostenson CG, Krarup T, Guerci B, et al Patients with Type 2 Diabetes Initiating Exenatide Twice Daily or Insulin in Clinical Practice: CHOICE Study. Diabetes Ther. 2012; 3:6 10.1007/s13300-012-0006-7 22714818 PMC3508107

[10] Fabunmi R, Nielsen LL, Quimbo R, Schroeder B, Misurski D, Wintle M et al Patient characteristics, drug adherence patterns, and hypoglycemia costs for patients with type 2 diabetes mellitus newly initiated on exenatide or insulin glargine. Curr Med Res Opin. 2009; 25: 777–786 10.1185/03007990802715199 19203299

[11] Becher H, Kostev K, Schröder-Bernhardi D. Validity and representativeness of the "Disease Analyzer" patient database for use in pharmacoepidemiological and pharmacoeconomic studies, Int J Clin Pharmacol Ther 2009; 47: 617–626 19825325 10.5414/cpp47617

[12] Quan H, Sundararajan V, Halfon P, Fong A, Burnand B, Luthi JC, et al Coding algorithms for defining co-morbidities in ICD-9-CM and ICD-10 administrative data. Med Care 2005; 43: 1130–1139. 16224307 10.1097/01.mlr.0000182534.19832.83

[13] Swart E, Gothe H, Geyer S, Jaunzeme J, Maier B, Grobe TG, et al Good Practice of Secondary Data Analysis (GPS): guidelines and recommendations. Gesundheitswesen. 2015; 77: 120–6. 10.1055/s-0034-1396815 25622207

[14] Busse R, Blümel M. Germany: Health system review. Health Syst Transit. 2014; 16: 1–296 25115137

[15] Rathmann W, Pscherer S, Konrad M, Kostev K. Diabetes treatment in people with type 2 diabetes and schizophrenia: Retrospective primary care database analyses. Prim Care Diabetes. 2015 4 27. [Epub ahead of print] 10.1016/j.pcd.2015.04.001 25937183

[16] Busse R. Disease management programs in Germanys statutory health insurance system. Health Affairs 2004; 23: 56–67 10.1377/hlthaff.23.3.56 15160803

[17] Tamayo T, Claessen H, Rückert IM, Maier W, Schunk M, Meisinger C, et al Treatment pattern of type 2 diabetes differs in two German regions and with patients' socioeconomic position. PLoS One. 2014; 9: e99773 10.1371/journal.pone.0099773 24915157 PMC4051778

